# Factorization machine with quadratic-optimization annealing for RNA inverse folding and evaluation of binary-integer encoding and nucleotide assignment

**DOI:** 10.1038/s41598-026-50891-7

**Published:** 2026-05-03

**Authors:** Shuta Kikuchi, Shu Tanaka

**Affiliations:** 1 Graduate School of Science and Technology, Keio University, Yokohama, Kanagawa 223-8522 Japan; 2 Keio University Sustainable Quantum Artificial Intelligence Center (KSQAIC), Keio University, Minato-ku, Tokyo 108-8345 Japan; 3 Department of Applied Physics and Physico-Informatics, Keio University, Yokohama, Kanagawa 223-8522 Japan; 4 Human Biology-Microbiome-Quantum Research Center (WPI-Bio2Q), Keio University, Minato-ku, Tokyo 108-8345 Japan

**Keywords:** Computational biology and bioinformatics, Mathematics and computing

## Abstract

The RNA inverse folding problem aims to identify nucleotide sequences that preferentially adopt a given target secondary structure. While various heuristic and machine learning-based approaches have been proposed, many require a large number of sequence evaluations, which limits their applicability when experimental validation is costly. We propose a method to solve the problem using a factorization machine with quadratic-optimization annealing (FMQA). FMQA is a discrete black-box optimization method reported to obtain high-quality solutions with a limited number of evaluations. Applying FMQA to the problem requires converting nucleotides into binary variables. However, the influence of integer-to-nucleotide assignments and binary-integer encoding on the performance of FMQA has not been thoroughly investigated, even though such choices determine the structure of the surrogate model and the search landscape, and thus can directly affect solution quality. Therefore, this study aims both to establish a novel FMQA framework for RNA inverse folding and to analyze the effects of these assignments and encoding methods. We evaluated all 24 possible assignments of the four nucleotides to the ordered integers (0-3), in combination with four binary-integer encoding methods. Our results demonstrated that one-hot and domain-wall encodings outperform binary and unary encodings in terms of the normalized ensemble defect value. In domain-wall encoding, nucleotides assigned to the boundary integers (0 and 3) appeared with higher frequency. In the RNA inverse folding problem, assigning guanine and cytosine to these boundary integers promoted their enrichment in stem regions, which led to more thermodynamically stable secondary structures than those obtained with one-hot encoding.

## Introduction

Ribonucleic acid (RNA) is an essential molecule that plays an important role in fundamental biological processes, including transcription and translation^1^, cellular differentiation and development^2^, catalyzing reactions^3^, and controlling gene expression^4^. In addition, synthetic RNAs are currently utilized in various fields, such as mRNA vaccines^5^, RNA aptamers^6^, genome editing^7^, biosensing^8^, ribozymes^9^, and riboswitches^10^.

**Fig. 1:**
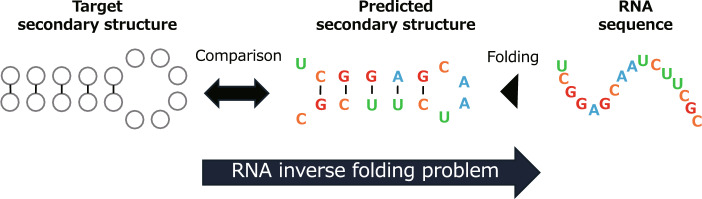
Overview of the RNA inverse folding problem. White circles represent arbitrary nucleotides in target secondary structure, and black lines indicate base pairs formed through hydrogen bonding. A, U, G, and C denote adenine, uracil, guanine, and cytosine, respectively.

The function of non-coding RNA (ncRNA) is largely determined by its structure. The primary structure of RNA refers to its linear sequence of four nucleotides linked by phosphodiester bonds. The nucleotides consist of a 5-carbon sugar ribose, a phosphate group, and one of four bases: adenine (A), uracil (U), guanine (G), and cytosine (C). Under physiological conditions, nucleotides in the sequence interact through specific hydrogen bonds to form canonical Watson–Crick base pairs (A-U, G-C)^11,12^ and, less commonly, wobble base pairs (U-G)^13^. Those base-pairing interactions lead to the creation of the secondary structure. Then, this secondary structure guides the formation of the three-dimensional structure, referred to as the tertiary structure. Although RNA function ultimately depends on its three-dimensional structure, which is determined by the underlying secondary structure arising from base-pairing interactions, synthetic RNAs can be readily constructed with arbitrary nucleotide sequences^14^. Consequently, the task of identifying RNA sequences that fold into a desired secondary structure (target structure) is known as the RNA inverse folding problem^15^ (Fig. 1). This problem is typically formulated as the search for an RNA sequence whose minimum free energy (MFE) structure matches the target structure. Theoretical results have shown that the problem is NP-hard, both in its most general formulation and even under simplified energy models^16,17^.

Several approaches have been developed to solve the RNA inverse folding problem^18^. The pioneering tool, RNAinverse^15^, uses an adaptive random walk to minimize the base pair distance between the MFE secondary structure of the current sequence and the target structure. Other approaches use stochastic local search^19–22^, genetic algorithms^23–25^, constraint programming^26^, ant colony optimization^27^, and Monte Carlo methods^28^. In addition, deep learning-based approaches have been proposed, including supervised learning from human solutions^29,30^, reinforcement learning^31,32^, and generative models^33^, and transformer-based deep representation learning^34^. These methods have advanced the field of the RNA inverse folding problem by achieving high success rates and producing high-quality sequences on difficult benchmark problems. However, the need for a large number of samples during the search process or for large-scale training data remains a limitation. Although digitally folded RNA sequences are commonly used for evaluation, wet-lab experiments benefit from approaches that limit the number of evaluations, given the considerable time and resource costs associated with experimental validation.

Black-box (BB) optimization methods that aim to reduce the number of expensive evaluations by combining surrogate modeling with iterative optimization have been actively studied^35–37^. These approaches construct a surrogate model from a limited number of observed samples and exploit it to efficiently search for better candidates, which leads to reduced overall evaluation cost. Recently, factorization machine with quadratic-optimization annealing (FMQA)^38^ has been proposed as a discrete black-box optimization method that employs a factorization machine (FM)^39^ as a surrogate model and performs optimization using quadratic optimization solvers. Originally, this framework is referred to as factorization machine with quantum annealing (FMQA)^40^. Ising machines are commonly used as quadratic optimization solvers. Ising machines have attracted attention as accurate and efficient solvers for combinatorial optimization problems^41^. Various types of Ising machines have been developed. Quantum annealing machines^42^ are implemented using superconducting circuits, and their internal algorithm is quantum annealing^43,44^. Digital Ising machines^45–48^ are implemented using digital circuits, such as graphics processing units (GPU), field-programmable gate arrays (FPGA). Their internal algorithms are based on simulated annealing (SA)^49,50^, simulated quantum annealing^51^, and simulated bifurcations^52^. To tackle a combinatorial optimization problem on an Ising machine, the problem must be formulated as a quadratic unconstrained binary optimization (QUBO) model. The QUBO model is given as

1 $$\begin{aligned} \mathscr {H}_{\textrm{QUBO}}(\{ \boldsymbol{x} \}) = \sum _{1\le i \le j \le N}Q_{i, j}x_{i}x_{j}, \end{aligned}$$

where $x_{i} \in \{0, 1\}$ and $Q_{i, j}$ is the (*i*, *j*)-th element of the *N*-by-*N* QUBO matrix *Q*. Thus, a limitation of the Ising machines has been the need to manually construct a suitable QUBO of the target model. However, in FMQA, this issue is handled by the mathematical equivalence between FM and QUBO. The FM is defined by

2 $$\begin{aligned} \mathscr {H}_\mathrm{{FM}}(\{ \boldsymbol{x} \}) = \omega _{0}+\sum _{i=1}^{N}\omega _{i}x_{i}+\sum _{1\le i < j \le N}\langle \boldsymbol{v}_i, \boldsymbol{v}_j \rangle x_{i}x_{j}, \end{aligned}$$

where $\omega _0 \in \mathbb {R}$, $\omega _{i} \in \mathbb {R}$, and $\boldsymbol{v}_{i} \in \mathbb {R}^K \ (i=1,\ldots ,N)$ are the model parameters. The parameter $K \in \mathbb {N}$ is the hyperparameter of the FM. The symbol $\langle \cdot , \cdot \rangle$ denotes the inner product. The FM model in Eq. (2) can be converted into a QUBO model. The diagonal elements of *Q* correspond to linear coefficients $\omega _i$, since $x_i x_j = x_i^2 = x_i$ when $i = j$, whereas the off-diagonal elements ($i \ne j$) are given by $\langle \boldsymbol{v}_i, \boldsymbol{v}_j \rangle$. Therefore, Ising machines can be applied even to problems where explicit QUBO modeling is difficult using FMQA. FMQA has been successfully applied to various domains, including material design^40,53–55^, polymer design^56,57^, engineering design^58,59^, feature selection^60,61^. Previous studies have reported that it can obtain high-quality solutions with fewer evaluations compared to random search, genetic algorithms, particle swarm optimization, and Bayesian optimization, which is a representative BB optimization method^40,53,57,59^.

In this study, we proposed a novel approach for applying FMQA to the RNA inverse folding problem. To tackle the problem using FMQA, it is necessary to convert nucleotides, which are categorical variables, into binary variables. Variables with more than two discrete states are commonly encoded as binary variables using binary-integer encoding. In contrast, a previous study employed a binary variational autoencoder (bVAE) to learn binary latent representations of amino acids^56^. The advantage of bVAE is its ability to efficiently convert a vast variety of variables, such as amino acids, while reducing the total number of required binary variables. However, since our study focuses on only four types of nucleotides, conventional binary-integer encoding is sufficient. Several types of binary-integer encoding exist, and a previous study has reported that the choice of encoding can significantly influence the performance of FMQA^62^. A previous study has demonstrated that an appropriate mapping to binary variables can reshape the energy landscape, reduce the probability of being trapped in local minima, and thereby improve the solution quality of FMQA^63^. It is also known that the specific encoding method affects the solution accuracy when solving problems with Ising machines^64–67^. While a previous report has analyzed the effects of converting ordered integers into binary variables^62^, there are no studies that analyze the role of binary-integer encoding when handling categorical variables within FMQA. Because nucleotides are categorical variables, their assignment to an integer is inherently arbitrary, and different binary-integer encoding methods may introduce distinct search biases. Therefore, this study investigates how binary-integer encodings and integer-to-nucleotide assignments influence solution quality in FMQA. Furthermore, we aim to evaluate the applicability of FMQA to the RNA inverse folding problem. The insights obtained here provide practical guidelines for applying FMQA to categorical optimization problems and lay the methodological foundation for its use in realistic RNA inverse folding scenarios. In this context, this work is intended as a methodological study of evaluation-efficient black-box optimization, rather than a benchmark study aimed at establishing a new state-of-the-art RNA inverse folding solver.

## Results

### Objective function of RNA inverse folding problem

The RNA inverse folding problem aims to identify an RNA sequence that preferentially adopts a target structure. In practical applications, the quality of an RNA sequence designed for a target secondary structure is evaluated by experimental validation. However, to evaluate the proposed method, we use the ensemble defect as a computationally evaluable objective function in this study.

The ensemble defect quantifies the expected number of nucleotides whose pairing status differs from that of the target secondary structure over the Boltzmann ensemble of RNA secondary structures. This metric evaluates not only the agreement between the MFE structure and the target structure, but also the thermodynamic stability of the target structure within the ensemble. Since its introduction by Dirks et al.^68^, ensemble defect has been adopted in several subsequent studies^21,22,26^. Dirks et al. demonstrated that ensemble defect outperforms structure distance based on MFE, achieving higher success rates in obtaining sequences that uniquely fold into the target structure^68^. More recently, Ward et al. confirmed that ensemble defect consistently performs better than structure distance between the MFE structure and target structure across diverse benchmarks^69^. Therefore, we used the ensemble defect.

An RNA sequence $\boldsymbol{n}$ of length *L* is represented by a string of nucleotides $n_{i} \in \{$ A, U, G, C $\}$. The secondary structure *s* of $\boldsymbol{n}$ is defined as a set of paired positions $(i, j) \ (i < j)$ such that $(n_{i}, n_{j})$ forms an allowed base-pair type (A-U, G-C, or U-G). Although natural RNAs may contain pseudoknots or higher-order interactions, these structural features are not supported by the folding model used here. Therefore, we consider only pseudoknot-free secondary structures.

Under the nearest-neighbor thermodynamic model^70^, each structure *s* of $\boldsymbol{n}$ is assigned a Gibbs free energy $\Delta G(\boldsymbol{n}, s)$. Assuming thermodynamic equilibrium at temperature *T*, the probability of observing *s* follows the Boltzmann distribution

3 $$\begin{aligned} p(s \ | \ \boldsymbol{n}) = \frac{\exp \!\left( -\Delta G(\boldsymbol{n}, s)/(RT)\right) }{Z(\boldsymbol{n})}, \end{aligned}$$

where *R* is the molar gas constant and

4 $$\begin{aligned} Z(\boldsymbol{n}) = \sum _{s \in S(\boldsymbol{n})} \exp \!\left( -\Delta G(\boldsymbol{n}, s)/(RT)\right) \end{aligned}$$

is the partition function, with $S(\boldsymbol{n})$ denoting the ensemble of all secondary structures that $\boldsymbol{n}$ can possibly fold into.

The ensemble defect is defined as the Boltzmann-weighted average structural distance

5 $$\begin{aligned} \phi (\boldsymbol{n}, t) = \sum _{s \in S(\boldsymbol{n})} p(s \ | \ \boldsymbol{n})\, d(s, t), \end{aligned}$$

where *d*(*s*, *t*) denotes the number of nucleotides whose pairing status differs between structure *s* and the target structure *t*. The normalized ensemble defect (NED), which ranges between 0 and 1, is given by

6 $$\begin{aligned} \textrm{NED}(\boldsymbol{n}, t) = \frac{\phi (\boldsymbol{n}, t)}{L}. \end{aligned}$$

The evaluation of ensemble defect was performed using the ViennaRNA package^71^.

### Proposed method

**Fig. 2:**
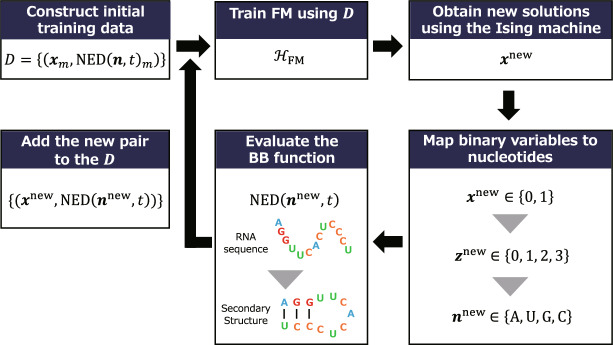
Schematic illustration of the proposed FMQA for the RNA inverse folding problem. As an example, the BB function is defined as the NED.

We proposed a novel approach to solve the RNA inverse folding problem based on FMQA. Figure 2 shows the procedure of the proposed method.

In practical applications, the BB function is typically assumed to correspond to experimentally measured properties, which may require substantial time and resources. However, to evaluate the applicability of FMQA in a controlled setting, we employed the computationally evaluable NED as the BB function in this study.

First, an initial training dataset $\{ (\boldsymbol{x}_{m}, \text {NED}(\boldsymbol{n}, t)_{m}) \} \ (m=1,\ldots ,M)$ is constructed by randomly generating solutions of binary variables $\boldsymbol{x}_{m}$ and their corresponding $\text {NED}(\boldsymbol{n}, t)_{m}$. We note that *M* is the number of initial data points. Here, the binary variables $\boldsymbol{x}$ are mapped to integer variables $\boldsymbol{z}$ using binary-integer encoding, and then the integer variables are mapped to nucleotides $\boldsymbol{n}$. Let *D* denote the dataset constructed from these data points.

Next, an FM is trained using the dataset *D*. The FM model is defined in Eq. (2). After training, new candidate solutions $\boldsymbol{x}^\text {new}$ are generated by optimizing an acquisition function using an Ising machine. In this study, the predicted cost output by the trained FM is directly employed as the acquisition function. The candidate solutions $\boldsymbol{x}^\text {new}$ are mapped to strings that represent the corresponding nucleotides $\boldsymbol{n}^\text {new}$. Then, the $\text {NED}(\boldsymbol{n}^\text {new}, t)$ are evaluated using the candidate solutions. The new pairs of solutions and their corresponding NEDs are appended to the dataset *D*.

This procedure, consisting of FM training, optimization using the Ising machine, and BB function evaluation, is repeated for a predefined number of iterations. Finally, the solution with the lowest ensemble defect observed in the dataset *D* is selected as the best solution obtained by FMQA.

### Binary-integer encoding method

In this study, we use four types of binary-integer encoding methods: one-hot, domain-wall, binary, and unary encoding. Since RNA consists of four types of nucleotides, each nucleotide can be represented as a discrete integer variable with four possible states. Table 1 presents the binary variable sequences corresponding to each integer $I \in \{$ 0, 1, 2, 3 $\}$ for the different binary-integer encoding methods. This subsection explains the binary representations of integers for each encoding method and their integration into FMQA.

**Table 1:** Binary variable sequence representing integers $I \in \{0, 1, 2, 3\}$ under different binary-integer encoding methods.

Integer	Binary variable sequence			
	One-hot encoding	Domain-wall encoding	Binary encoding	Unary encoding
0	1000	000	00	000
1	0100	100	10	100, 010, 001
2	0010	110	01	110, 101, 011
3	0001	111	11	111

One-hot encoding is a traditional method. An integer is represented by the position of a single binary variable that takes the value 1. An integer *I* is given by $I = \sum _{i = 0}^{3} i x_{i}$. To handle the one-hot encoding into FMQA, a penalty term is added to the FM model in Eq. (2). The model is given as

7 $$\begin{aligned} \mathscr {H}_{\textrm{FM}}^{\textrm{oh}} = \mathscr {H}_{\textrm{FM}} + \mu \sum _{l = 1}^{L} \left( \sum _{i = 0}^3 x_{i}^{(l)} - 1 \right) ^ 2, \end{aligned}$$

where $\mu > 0$ is a penalty coefficient that controls the strength of the penalty term, and $x_{i}^{(l)}$ represents binary variables for an *l*-th integer variable. Domain-wall encoding was originally proposed by Chancellor^64^. An integer is represented by the number of leading binary variables that take the value 1, followed by 0s. An integer is given by $I = \sum _{i = 0}^{2} x_{i}$. The boundary position between the binary variables set to 1 and those set to 0 is referred to as a domain wall. To enforce the constraint that exactly one domain wall exists, a penalty term is added to the FM model. The extended FM model is given as

8 $$\begin{aligned} \mathscr {H}_{\textrm{FM}}^{\textrm{dw}} = \mathscr {H}_{\textrm{FM}} + \mu \sum _{l = 1}^{L} \sum _{i = 0}^1 x_{i+1}^{(l)} (1 - x_{i}^{(l)}). \end{aligned}$$

Binary encoding uses the binary representation for encoding integers. An integer *I* is given by $I = \sum _{i = 0}^{1} 2^{i} x_{i}$. In the RNA inverse folding problem, binary encoding does not require an additional penalty term, as each binary configuration uniquely corresponds to an integer value. Unary encoding represents an integer by the number of binary variables set to value 1, similar to domain-wall encoding. Since this representation does not impose an explicit structural constraint, no penalty term is required. Unary encoding admits multiple binary representations for a single integer value. When applying FMQA, the total number of binary variables depends on the chosen encoding method. One-hot encoding requires $N = 4L$, domain-wall and unary encodings require $N = 3L$, and binary encoding requires $N = 2L$.

A previous study investigated the effect of binary-integer encoding methods on FMQA. One-hot, domain-wall, and binary encoding, were evaluated using the ground-state energy calculation of a hydrogen molecule^62^. The problem was originally formulated with real-valued variables, and these variables were discretized using binary-integer encoding. While all encoding methods achieved high accuracy, one-hot and domain-wall encodings outperformed binary encoding in terms of energy error distribution and stability. In particular, one-hot encoding consistently yielded low energy errors even with small *K*.

### Evaluation of effect of binary-integer encoding method and integer-to-nucleotide assignment

To investigate the conditions under which FMQA can effectively solve the RNA inverse folding problem, we investigated the effects of binary-integer encoding and integer-to-nucleotide assignment. For binary-integer encoding, we considered one-hot, domain-wall, binary, and unary encodings. For integer-to-nucleotide assignment, we evaluated all possible $4! = 24$ assignments of the four nucleotides to the integers (0, 1, 2, 3). For each condition, the optimization was performed independently 10 times.

The objective function was the NED. Since the NED represents the structural error with respect to the target structure, the problem is formulated as a minimization task. The number of FMQA iterations was set to 1000, and the number of initial training data points was set to 10. We used an SA-based Ising machine^48^, which runs on a GPU. Other parameters are described in the “Methods” section. As the target structure, we used *stickshift* from the well-known benchmark Eterna100^72^. This structure consists of 26 nucleotides (nt) and has a relatively simple secondary structure. Thus, it provides an appropriate test case for evaluating the effects of encoding and assignment.

**Fig. 3:**
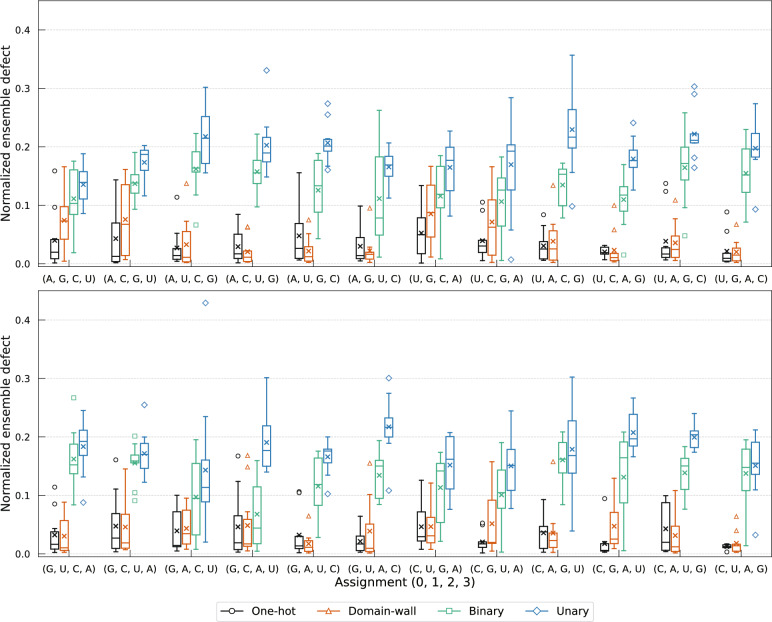
Normalized ensemble defect values obtained by FMQA under different combinations of binary-integer encoding methods and integer-to-nucleotide assignments. For each assignment, the encodings are shown from left to right as one-hot, domain-wall, binary, and unary encodings. Crosses indicate the average NED over 10 runs. The upper and lower whiskers denote the maximum and minimum NED values, respectively. Black circle, orange triangle, green square, and blue diamond represent outliers. Panels in the top row correspond to assignments in which A or U was assigned to integer 0, whereas panels in the bottom row correspond to assignments in which G or C was assigned to integer 0.

Figure 3 shows the solutions obtained by FMQA under different combinations of binary-integer encoding methods and integer–to-nucleotide assignments. The ensemble defect corresponds to the expected number of nucleotides whose pairing status differs from that of the target secondary structure under the Boltzmann ensemble of possible structures. Therefore, a lower NED indicates that the sequence more reliably adopts the target structure. Focusing on the binary-integer encoding methods, one-hot and domain-wall encodings achieved lower NED values compared with binary and unary encodings. Binary encoding yielded lower NED values than unary encoding. With respect to integer–to-nucleotide assignment, the ensemble defect values of domain-wall encoding were relatively high for the assignments (A, G, C, U), (A, C, G, U), (U, G, C, A), and (U, C, G, A). In contrast, one-hot encoding consistently achieved low NED values regardless of the assignment.

**Fig. 4:**
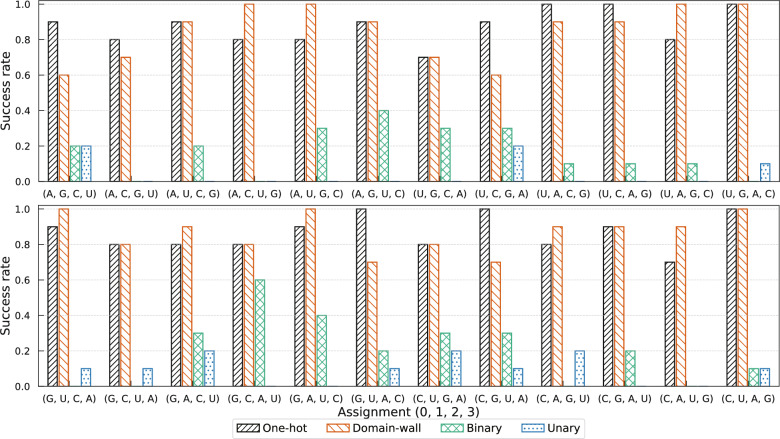
Success rate obtained by FMQA under different combinations of binary-integer encoding methods and integer-to-nucleotide assignments. For each assignment, the encodings are shown from left to right as one-hot, domain-wall, binary, and unary encodings. Panels in the top row correspond to assignments in which A or U was assigned to integer 0, whereas panels in the bottom row correspond to assignments in which G or C was assigned to integer 0.

The objective of the RNA inverse folding problem is to identify RNA sequences whose MFE secondary structure coincides with the target structure. Therefore, we computed the MFE structure for each RNA sequence obtained by FMQA and examined whether it matched the target structure. Solutions whose MFE structures were identical to the target structure were defined as success solutions, and the proportion of success solutions among the 10 runs was defined as the success rate. The success rates under each condition are shown in Fig. 4.

Binary and unary encodings exhibited relatively high NED values, and consequently, their success rates were low, with some conditions yielding a success rate of 0.0. In contrast, one-hot and domain-wall encodings generally achieved high success rates. However, for the assignments in which domain-wall encoding showed high NED values, the success rate was correspondingly low.

**Fig. 5:**
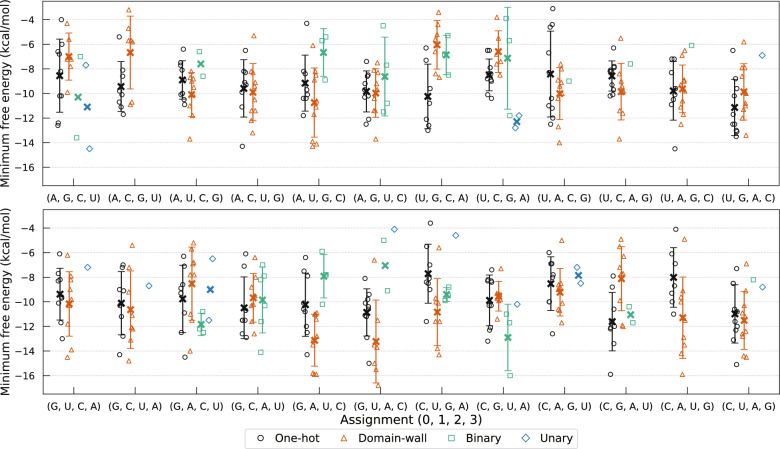
Minimum free energy values obtained by FMQA under different combinations of binary-integer encoding methods and integer-to-nucleotide assignments. For each assignment, the encodings are shown from left to right as one-hot, domain-wall, binary, and unary encodings. Only success solutions are plotted. When two or more success solutions were obtained, their average MFE value is indicated by a cross marker. When three or more success solutions were obtained, the standard deviation is shown as error bars. Panels in the top row correspond to assignments in which A or U was assigned to integer 0, whereas panels in the bottom row correspond to assignments in which G or C was assigned to integer 0.

Finally, we analyzed the MFE values of the success solutions. The MFE values of the success solutions are presented in Fig. 5. One-hot encoding yielded solutions with comparable MFE values (approximately $-10$ kcal/mol) across all assignments. In contrast, domain-wall encoding exhibited assignment-dependent behavior. For the assignments (A, G, C, U), (A, C, G, U), (U, G, C, A), and (U, C, G, A), where the NED values were high, the corresponding MFE values were also relatively high. Conversely, for the assignments (G, A, U, C), (G, U, A, C), (C, A, U, G), and (C, U, A, G), the MFE values were lower compared to those obtained with other assignments.

### Comparison of the number of BB function evaluations

In the previous subsection, we analyzed the effects of binary-integer encoding and nucleotide assignments from the viewpoint of final solutions obtained by FMQA. In this subsection, we compare FMQA with other black-box optimization methods in terms of the number of objective function evaluations. In practical applications, each BB function evaluation may correspond to a wet-lab experiment, which typically requires a substantial amount of time. In such settings, the computational time of surrogate training and optimization is negligible compared to the time required for wet-lab experimentation. Therefore, the number of BB function evaluations is the primary performance metric.

Although several methods have been proposed for the RNA inverse folding problem^15,19–28^, their objective functions often differ from that used in our study, and in many cases the number of objective function evaluations cannot be directly measured or fairly compared. Therefore, in this study, we compared the number of evaluations using Bayesian optimization and a genetic algorithm (GA), both using NED as the objective function.

For Bayesian optimization, we employed the tree-structured Parzen estimator (TPE), which can directly handle categorical variables. For FMQA, we used the integer-to-nucleotide assignment (G, A, U, C), which achieved a success rate of 1.0, low MFE values, and the lowest NED among the domain-wall encoding results in the previous subsection. The results obtained with one-hot and domain-wall encodings under this assignment were used for comparison. In addition, random search (RS) was adopted as a baseline method. All methods were initialized with the same initial dataset as FMQA to ensure a fair comparison.

**Fig. 6:**
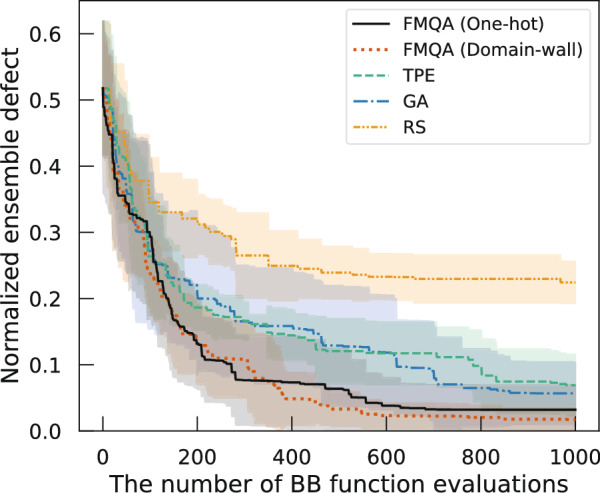
Relationship between the number of BB function evaluations and the best normalized ensemble defect value up to each evaluation.

Figure 6 shows the relationship between the number of NED evaluations and the best NED value obtained up to each evaluation for each method. Each method was independently performed 10 times, and the mean and standard deviation across runs are presented. As shown in Fig. 6, FMQA achieved lower NED values with fewer evaluations compared to the other methods. The result demonstrates its efficiency in reducing the number of expensive objective function evaluations in the RNA inverse folding problem.

### Performance on multiple target secondary structures

In this subsection, we evaluated the performance of FMQA on target secondary structures other than *stickshift*.

**Fig. 7:**
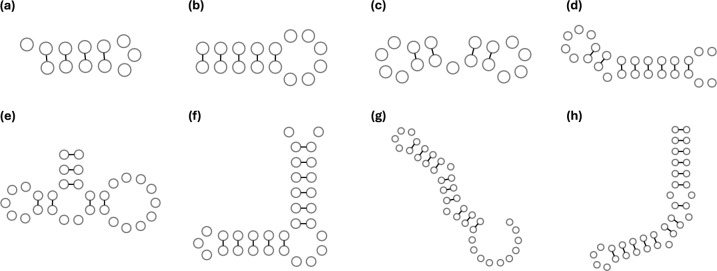
Target secondary structures selected from the Eterna100 benchmark and evaluated in this study. White circles represent nucleotides, and black lines indicate base pairs formed by hydrogen bonding. The target structures are: (**a**) *G-C Placement* (12 nt), (**b**) *Simple Hairpin* (16 nt), (**c**) *Shortie 4* (17 nt), (**d**) *stickshift* (26 nt), (**e**) *Small and Easy 6* (30 nt), (**f**) *Corner bulge training* (31 nt), (**g**) *Prion Pseudoknot – Difficulty Level 0* (36 nt), and (**h**) *infoRNA test 16* (36 nt).

From the Eterna100 benchmark, we selected eight structures with relatively small nucleotide lengths (12–36 nt). The selected target structures are shown in Fig. 7.

FMQA was performed using the integer-to-nucleotide assignment (G, A, U, C), which demonstrated better performance in the previous subsection. We compared one-hot and domain-wall encoding under the same experimental conditions. For each target structure, the optimization was independently executed 10 times.

**Fig. 8:**
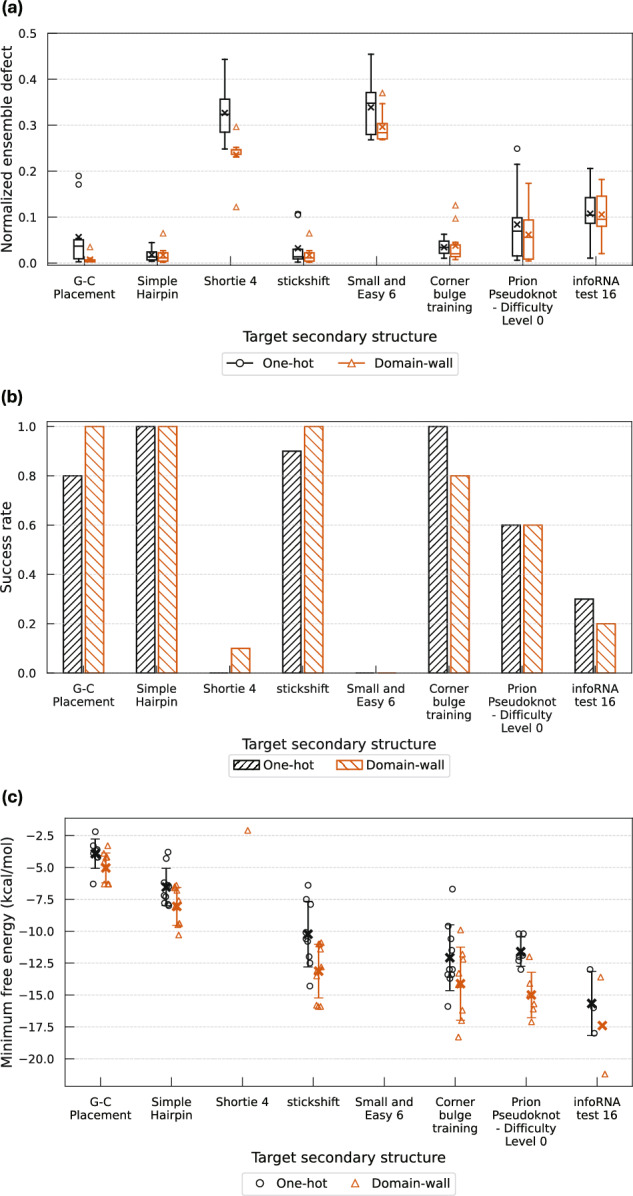
Performance metrics on multiple Eterna100 benchmark target structures. For each assignment, the encodings are shown from left to right as one-hot and domain-wall encodings. (**a**) Normalized ensemble defect values obtained by FMQA for each target structure. Crosses indicate the average NED over 10 independent runs. The upper and lower whiskers denote the maximum and minimum NED values, respectively. Black circle and orange triangle represent outliers. (**b**) Success rate. (**c**) Minimum free energy values. Only success solutions are plotted. When two or more success solutions were obtained, their average MFE value is indicated by a cross marker. When three or more success solutions were obtained, the standard deviation is shown as error bars.

Figure 8(a) presents the NED obtained for each structure. Overall, domain-wall encoding achieved lower NED values than one-hot encoding for most target structures. In particular, for *G-C Placement*, *Simple Hairpin*, *stickshift*, and *Corner bulge training*, both encoding methods consistently yielded low NED values across runs. In contrast, *Shortie 4* and *Small and Easy 6* showed relatively higher NED values. The corresponding success rates are shown in Fig. 8(b). Except for *Shortie 4* and *Small and Easy 6*, at least two success solutions were obtained for each structure using both encoding methods. Notably, for *G-C Placement, Simple Hairpin, stickshift*, and *Corner bulge training*, the success rate was high for both encodings. In contrast, *Prion Pseudoknot – Difficulty Level 0* and *infoRNA test 16* exhibited lower success rates compared with their target structures. Figure 8(c) shows the MFE values of the success solutions. Consistent with the NED, domain-wall encoding achieved lower MFE values than one-hot encoding for most target structures.

## Discussion

In this study, we demonstrated that, in the RNA inverse folding problem solved by FMQA, one-hot and domain-wall encodings achieved better solutions than binary and unary encodings. This is consistent with a previous study that investigated the effect of binary-integer encoding methods on FMQA when continuous variables were discretized into binary variables^62^. In that study, the energy error, defined as the difference between the optimal objective value and the value obtained by FMQA, was smaller for one-hot and domain-wall encodings than for binary encoding. In contrast, a previous study on the relationship between Ising machines and binary-integer encodings reported that binary and unary encodings sometimes outperformed one-hot encoding in terms of feasibility rate and objective value quality^65^. These findings suggest that, in FMQA, the choice of binary-integer encoding primarily influences solution quality through the construction and expressivity of the FM, rather than through the optimization performance of the Ising machine. Binary encoding represents four nucleotides using only two binary variables. Although this representation is compact, it may limit the expressive capacity of the FM to model complex nonlinear interactions among categorical states. For unary encoding, multiple binary configurations correspond to the same integer value. This redundancy increases representational degeneracy. The FM must implicitly learn that these distinct binary configurations correspond to the same nucleotide, which may complicate surrogate modeling and reduce optimization efficiency. Therefore, the observed degradation in solution quality for unary encoding may be attributed to the inability of the FM to properly capture this many-to-one mapping structure.

**Fig. 9:**
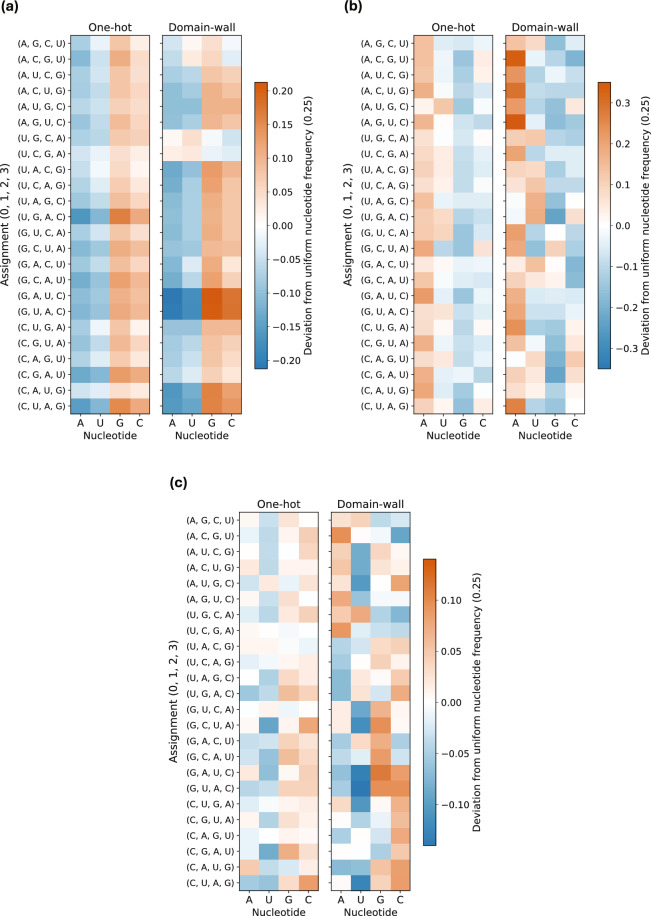
Nucleotide frequencies obtained from the success solutions across different integer-to-nucleotide assignments. (**a**) Nucleotide frequency in stem regions, (**b**) nucleotide frequency in non-stem regions, and (**c**) nucleotide frequency over the entire RNA sequence. The baseline value of 0.25 corresponds to the expected frequency under a random uniform nucleotide frequency. Orange indicates frequencies higher than 0.25, whereas blue indicates frequencies lower than 0.25.

We next discuss the effect of integer–to-nucleotide assignment. In domain-wall encoding, certain assignments resulted in different NED and MFE values. To analyze these results, we investigated nucleotide frequency in the solutions obtained under all assignments. Based on the secondary structure, nucleotides were categorized into stem regions (base-paired positions) and non-stem regions (unpaired positions). Nucleotide frequencies were calculated by pooling all nucleotides from the successful solutions obtained in 10 independent runs and normalizing by the total number of nucleotides. The results are shown in Fig. 9. Figure 9(a) shows nucleotide frequency in stem regions, Fig. 9(b) in non-stem regions, and Fig. 9(c) across the entire sequence. The baseline value of 0.25 corresponds to uniform nucleotide frequency. The heatmap shows the deviation of nucleotide frequency from this uniform expectation.

In stem regions (Fig. 9a), one-hot encoding consistently exhibited high G and C frequency across all assignments. This observation agrees with previous reports on RNA inverse folding^19,20,25,68^. G-C base pairs form three hydrogen bonds and are typically more stable than A-U or U-G pairs. The stability of the structure is determined by nearest-neighbor stacking interactions between adjacent base pairs. In the Turner thermodynamic model implemented in the ViennaRNA package, G-C stacks exhibit the most favorable free energies, explaining why enrichment of G-C pairs in stem regions lowers MFE and reduces ensemble defect^70,73^. In contrast, domain-wall encoding exhibited assignment-dependent results. In assignments where A-U and U-G pairs were more prevalent in stems, NED and MFE values were higher: (A, G, C, U), (A, C, G, U), (U, G, C, A), and (U, C, G, A). These assignments correspond to those that showed inferior optimization performance. Since A-U and U-G pairs are thermodynamically less stable than G-C pairs, enrichment of these pairs in stems increases free energy and reduces structural stability. Meanwhile, assignments in which G-C frequency in stems exceeded that of one-hot encoding corresponded to lower MFE values, (G, A, U, C), (G, U, A, C), (C, A, U, G), and (C, U, A, G). This further supports the interpretation that stem thermodynamic stability directly influences FMQA performance in RNA inverse folding.

In non-stem regions (Fig. 9(b)), one-hot encoding showed a high frequency of adenine, consistent with the results of previous methods^25^. Adenine forms canonical base pairs only with uracil. Compared with guanine, which can pair with both cytosine and uracil, adenine reduces the likelihood of unintended base pairing. Although cytosine also pairs with only guanine, erroneous C-G pairing is energetically more stabilizing than A-U pairing. Therefore, placing adenine in loop regions reduces the risk of forming undesired stable alternative structures, thereby lowering the ensemble defect. In domain-wall encoding, adenine frequency remained relatively high in non-stem regions. However, the frequency of other nucleotides varied substantially depending on assignment. Notably, nucleotides assigned to integer 0 tended to exhibit higher frequency.

When examining overall nucleotide frequency (Fig. 9c), domain-wall encoding showed increased frequency for nucleotides assigned to integers 0 and 3. This phenomenon can be interpreted from the viewpoint of Hamming distance between binary representations. In one-hot encoding, the Hamming distance between any pair of integers is uniformly two. In contrast, domain-wall encoding exhibits asymmetric distances. Adjacent integers differ by Hamming distance one, whereas non-adjacent integers differ by two or three binary variables. Adjacent integers are directly connected within the feasible subspace, while non-adjacent integers are separated by larger Hamming distances^67^. In domain-wall encoding, integers 0 and 3 each have only one neighboring integer, whereas middle integers (1 and 2) have two such neighbors. This asymmetry may bias the search dynamics, making transitions away from 0 and 3 less frequent. As a result, nucleotides assigned to boundary integers 0 and 3 may appear more frequently in the optimized sequences.

In the present study, we did not explicitly constrain GC content or impose thermodynamic priors beyond ensemble defect minimization. Therefore, achieving low free energy is central to success in the RNA inverse folding problem considered here. The results suggest that, when using domain-wall encoding, careful design of integer–to-nucleotide assignment can exploit encoding-specific search biases. For example, assigning G and C to integers whose binary representations are less prone to transition may enhance stem stability and reduce free energy. Thus, domain-wall encoding provides not only a representation method but also an opportunity for encoding-aware problem design.

We discuss the influence of target secondary structure on FMQA performance. In the RNA inverse folding problem, it has been reported that structures containing very short stems (e.g., two base pairs) flanked by unpaired nucleotides are particularly difficult to design^72^. Such short stems are thermodynamically unstable and can be easily disrupted by alternative competing structures, making it difficult to identify sequences that uniquely fold into the target structure. Among the target structures evaluated in this study, *Shortie 4* and *Small and Easy 6*, for which almost no success solutions were obtained, exhibit this characteristic feature. Our results indicate that FMQA also struggles with these inherently unstable structural motifs. In contrast, bulge-loop structures, where one strand of a continuous helix contains unpaired nucleotides, are also known to increase design difficulty because they introduce asymmetry and local flexibility. Nevertheless, FMQA successfully obtained solutions for *Corner bulge training*, which contains such bulge motifs. This result suggests that FMQA can effectively handle certain classes of structural irregularities, provided that the overall stem length remains sufficiently long to ensure thermodynamic stability. Furthermore, even when a structure contains multiple stable stems, increasing the total nucleotide length expands the combinatorial search space exponentially. As sequence length increases, the number of possible nucleotide combinations grows as $4^L$, thereby increasing the complexity of the optimization landscape. Consequently, the 36-nt structures (*Prion Pseudoknot – Difficulty Level 0* and *infoRNA test 16*) exhibited lower success rates compared with shorter targets. This reduction in performance can be attributed not only to structural complexity but also to the enlarged search space associated with longer sequences. These observations indicate that FMQA performance is influenced by both the thermodynamic stability of local motifs and global combinatorial complexity. Addressing these difficulties may require the introduction of explicit base-pairing constraints or the adoption of motif-level decomposition strategies explored in previous RNA inverse folding studies^19,68^. Several previous approaches to RNA inverse folding have reported high success rates for target secondary structures similar to those considered in this study^32,74^. Direct comparison is not straightforward due to differences in objective functions, particularly because this study employs ensemble defect. Nevertheless, comparisons based on alternative metrics, such as success rate or the number of solved target secondary structures in Eterna100, would still be informative. Future research will focus on extending the applicability of FMQA to a broader class of RNA inverse folding problems and systematically comparing its performance with established approaches^15,19–34^.

Finally, we note that the evaluation in this study is limited to a thermodynamic framework based on the nearest-neighbor energy model. While this approach ensures methodological consistency, further evaluation using alternative BB functions would be valuable. This would allow us to evaluate the influence of encoding methods and assignments on model performance beyond the nearest-neighbor energy model, and to strengthen the biological relevance of the obtained solutions. For example, as suggested in previous work^75^, such validation could be performed by predicting the three-dimensional structures of the designed sequences, deriving their corresponding secondary structures, and evaluating their agreement with the target structures.

## Methods

### RNA secondary structure analysis

RNA secondary structure prediction and ensemble evaluation were performed using the ViennaRNA package (v2.7.2)^71^. All calculations were conducted at a constant temperature of 37$^\circ$C to reflect physiological conditions. This temperature corresponds to the default setting of the package and has been widely adopted in previous RNA folding studies^15,22,25,69^. For the energy model, the dangle treatment was set to 2 (–dangles=2). This setting accounts for the energetic contributions of dangling ends and terminal mismatches, approximating coaxial stacking effects and providing a thermodynamically consistent estimation of structural stability.

### FMQA settings

**Fig. 10:**
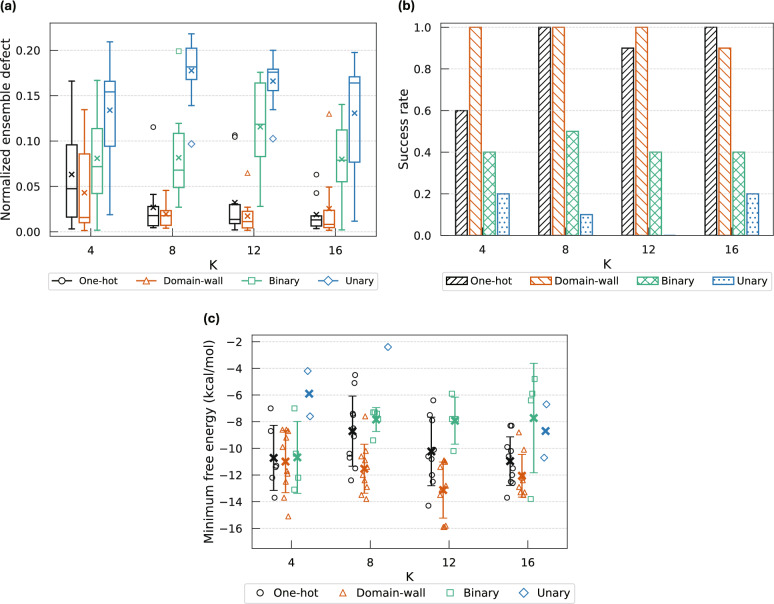
Performance metrics for different values of the hyperparameter *K* under each binary-integer encoding method. For each assignment, the encodings are shown from left to right as one-hot, domain-wall, binary, and unary encodings. (**a**) Normalized ensemble defect values obtained by FMQA for each encoding method and value of *K*. Crosses indicate the average NED over 10 independent runs. The upper and lower whiskers denote the maximum and minimum NED values, respectively. Black circle, orange triangle, green square, and blue diamond represent outliers. (**b**) Success rate. (**c**) Minimum free energy values of the success solutions. Only success solutions are plotted. When two or more success solutions were obtained, their average MFE value is indicated by a cross marker. When three or more success solutions were obtained, the standard deviation is shown as error bars.

The FM was trained using the AdamW optimizer with a learning rate of 0.01. The number of training epochs was set to 1000, and the mean squared error (MSE) was used as the loss function. The hyperparameter *K* was determined based on a comparative performance analysis. We evaluated $K = 4, 8, 12,$ and 16 for each binary-integer encoding method. The integer-to-nucleotide assignment was fixed to (G, A, U, C). For each condition, the optimization was independently performed 10 times. The results are shown in Fig. 10. Figure 10(a) presents the NED values. One-hot and domain-wall encodings exhibited low NED values for all *K* except $K = 4$. Binary encoding showed slight performance degradation at $K = 12$, and unary encoding at $K = 8$. Across all *K*, the order of performance was consistent: one-hot and domain-wall encodings achieved the lowest NED values, followed by binary encoding, and finally unary encoding. The success rates are shown in Fig. 10(b). One-hot encoding exhibited a lower success rate at $K = 4$, whereas domain-wall encoding maintained a high success rate across all *K*. Figure 10(c) shows the MFE values of the obtained success solutions. For one-hot encoding, the MFE values at $K = 8$ were slightly higher than those at other *K* values. In domain-wall encoding, the lowest MFE values were observed at $K = 12$. Based on these results, and considering the performance of one-hot and domain-wall encodings, we selected $K = 12$ for the FMQA experiments reported in the “Results” section.

The trained FM model was solved using an SA-based Ising machine^48^ implemented on a GPU. The limit of computation time per optimization run was set to 2000 ms. Before solving the FM model on the Ising machine, all coefficients were normalized by dividing them by the maximum absolute coefficient value. For one-hot and domain-wall encodings, a penalty coefficient was set to $\mu = 2$. During the FMQA iterations with this penalty coefficient, no infeasible solutions were observed. At each FMQA iteration, the Ising machine generated one new candidate solution $\boldsymbol{x}^{\textrm{new}}$, which was evaluated to evaluate the NED and appended to the dataset.

The initial dataset consisted of 10 randomly generated sequences. Each sequence was constructed by randomly selecting nucleotides from the set $\{A, U, G, C\}$, with the sequence length equal to that of the target secondary structure. FMQA was performed for 1000 iterations. The best solution observed during the optimization process was selected as the final output of FMQA.

The above settings were kept identical across all target secondary structures.

### Baseline optimization methods

Random search (RS) and tree-structured Parzen estimator (TPE) were implemented using the Optuna package. For TPE, the multivariate parameter was set to True to enable multivariate sampling of categorical variables.

The genetic algorithm (GA) was implemented using the pymoo package^76^, which has been used in previous comparative studies with FMQA^57^. The GA was formulated as a single-objective optimization problem. Integer variables representing nucleotides were optimized directly in the range $\{0,1,2,3\}$. The population size was set to 10, identical to the initial dataset size used in FMQA to ensure fair comparison. Uniform crossover was employed with crossover probability 0.9. Mutation was implemented as a random-reset mutation operator, where each gene was mutated with probability $p = 5.0 / L$, with *L* denoting the sequence length (for stickshift, $L = 26$). When mutation occurred, the nucleotide value was randomly reassigned to a different integer. The termination criterion was based on the total number of objective function evaluations. The evaluation budget was set to 1000 objective function evaluations, excluding the evaluations of the initial dataset, to match the number of additional NED evaluations performed in FMQA.

All methods were initialized with the same initial dataset as FMQA.

## Data Availability

The datasets used in our study are available from the corresponding author upon reasonable request.

## Code availability

The implementation is available at: https://github.com/Shu-Tanaka-Group/fmqa_rna.
